# Prenatal Exposure to Antiepileptic Drugs and Dental Agenesis

**DOI:** 10.1371/journal.pone.0084420

**Published:** 2014-01-08

**Authors:** Pernille E. Jacobsen, Tine B. Henriksen, Dorte Haubek, John R. Østergaard

**Affiliations:** 1 Section of Pediatric Dentistry, Department of Dentistry, Health, Aarhus University, Aarhus, Denmark; 2 Perinatal Epidemiology Research Unit, Department of Pediatrics, Aarhus Universty Hospital, Aarhus, Denmark; 3 Center for Rare Diseases, Department of Pediatrics, Aarhus University Hospital, Aarhus, Denmark; University of Toronto, Canada

## Abstract

**Objective:**

The aim of the study was to investigate the association between prenatal exposure to AEDs and the risk of dental agenesis and to differentiate between the possible effects of the different drugs used.

**Methods:**

Data on 214 exposed and 255 unexposed children, aged 12–18 years, were extracted from the Prescription Database of the Central Denmark Region and North Denmark Region and the Danish Medical Birth Registry. The children's dental charts were examined for the presence of dental agenesis.

**Results:**

Overall, children exposed to AED *in utero* had an increased risk of developing dental agenesis, but as a group, the difference was not significant (OR = 1.7; [95% CI: 0.8–3.6]). The risk of developing dental agenesis was three-fold increased (OR = 3.1; [95% CI: 1.3–7.4]) in children exposed to valproate in mono- or in poly-therapy with other AEDs than carbamazepine or oxcarbazepine. The risk was further increased (OR = 11.2; [95% CI: 2.4–51.9]) in children exposed to valproate and carbamazepine or oxcarbazepine in combination.

**Conclusions:**

The present study shows that dental agenesis is a potential congenital abnormality that is related to prenatal exposure to valproate, and dental agenesis may be considered a sensitive marker for the teratogenicity of valproate.

## Introduction

The most commonly reported congenital malformations in children exposed *in utero* to anti-epileptic drugs (AED) are mid-face hypoplasia, digital hypoplasia, and neural tube defects [1], [2]. Children exposed to valproate *in utero* may develop ‘fetal valproate syndrome’, which is characterized by facial features like a flat nose, a broad nasal root, and shallow philtrum in addition to major congenital malformations [3]. Various known genetic syndromes with cranio-facial deformities like Down syndrome, Rieger's syndrome, and lacrimo-auriculo-dento-digital syndrome [4] are all associated with dental abnormalities. To our knowledge, this association has never been reported in cases of ‘fetal valproate syndrome’, although congenital deformation that involves the mid-face section is known to carry a high risk of concomitant dental abnormalities within the same developmental area [5].

Non-syndromic dental agenesis, of the permanent teeth, is the most common congenital malformation in man [6]. The reported prevalence varies worldwide, and the estimated prevalence among Caucasians in Europe is 5.5% [7]. Non-syndromic dental agenesis is often heritable, as shown in numerous of family and twin studies [8], [9], but can also arise due to postnatal exogenous exposures as demonstrated in children undergoing cancer therapy [10] and children exposed to high levels of dioxin [11]. In these cases, the condition called dental aplasia because the development of the tooth is arrested and the tooth germ is reabsorbed. Dental agenesis of the primary teeth is a rare condition and the estimated prevalence in Europe varies from 0.2–0.5% [12], [13]. Agenesis of primary teeth is almost always associated with agenesis of the equivalent permanent tooth [14], [15], but has to our knowledge never been associated with external harmful exposures.

Very few studies have investigated the incidence of dental agenesis of permanent teeth due to prenatal exposure to AED, and the findings are contradictory [16], [17]. In a recently published study, we showed that children exposed to AED *in utero* had an increased risk of developing enamel defects in both the primary and the permanent teeth [18]. These results indicate that the different stages in the amelogenesis are sensitive to AED exposure. However, it is unknown if it also influences the genetic expression and cause dental agenesis. The aim of the present study was to investigate the risk of dental agenesis of the permanent teeth in children prenatally exposed to AED and to elucidate the association of such an exposure to other congenital abnormalities.

## Materials and Methods

The study was registered as a registry-based study and approved by the Danish National Board of Health (7-604-04-2/140/EHE), and registered and approved by the Danish Data Protection Agency. These approvals allow us to use information's from the databases without a written consent from the parents of the children included in the study.

The study was conducted as a cohort-based study with prospective collection of information from the Prescription Database of the Central Denmark Region and North Denmark Region and the Danish Medical Birth Registry. The former contains information on prescriptions for refundable drugs in Denmark, such as type of drug (Anatomical Therapeutic Classification (ATC) coding), dose, and package size, among others [19]. The Prescription Database contains data on inhabitants living in the Northern and Central Denmark Region, i.e. about one-third of the Danish population. The Danish Medical Birth Registry has prospectively collected data on all newborns in Denmark since 1973. The registry comprises birth-related information such as gestational age at birth (GA), birth weight (BW), and Apgar score, among others. The registry also contains information about the mother, e.g. smoking habits and age at birth [20]. Information was extracted on smoking during pregnancy and newborn characteristics for children known from the Prescription Database to have been exposed to AED during pregnancy. In total, 232 children were considered exposed. The children were between 12–18 years of age when enrolled into the study. We estimated that the number of controls should be approximately the same as the number of the exposed children. This was based on an estimate of a three times higher prevalence rate of 15% in exposed than in unexposed children, a statistical significance level defined as a two-sided *p*-value below 5%, and a study power of 100%. Controls within the same age range without exposure *in utero* to AED were also located in the Danish Medical Birth Registry. The entire study base comprised 469 children. Five children had passed away and 12 children had emigrated. The number of drop-outs was equally distributed between the exposed and unexposed group. The reasons for drop-out are listed in Table 1.

**Table 1:** Elimination process prior to analysis.

Exclusion criteria	Number of exposed children	Number of unexposed children
Study base	232	272
Diseased	2	3
Emigrated	7	5
No response from dentist	5	5
Incomplete dental chart	4	4
Total enrolled in the study	214	255

The addresses of all children (exposed children and controls) were obtained from the Civil Registration Registry. The data were subsequently alphabetically organized, first by the participant's home municipality and, second, by his or her first name in order to ensure total blindness in the recording of the dental investigations.

In Denmark, all children are offered free dental service until the age of 18, and registration of dental agenesis is an obligatory part of the orthodontic visitation at the age of 12 years [21]. Therefore, all children are clinically examined by a dentist or orthodontist at this particular age and dental agenesis of permanent teeth is recorded in the child's dental chart. In case of early exfoliation of primary teeth or persistence of permanent teeth, the chart will contain radiographs verifying the existence of the permanent teeth. In order to gain access to the individual child's dental charts, the chief dentists of the children's home-town were contacted by letter. In case of no response, a reminder was sent out. The dental charts were all examined by the first author to verify the presence of dental agenesis. Dental agenesis was only recorded if the permanent tooth was already erupted into the oral cavity or radiologically verified. In case of incomplete information, the community dental services was informed, and the charts were re-examined approximately one year later than the first examination. The outcome was recorded as “0” for no agenesis, “1” for agenesis, “2” for extracted due to orthodontic treatment, and “9” if not recorded. Possible agenesis of third molar was not included.

In order to elucidate any possible correlation between dental agenesis and other congenital abnormalities, data on congenital malformations were extracted from the Danish National Registry of Patients [22]. This registry holds data on all diagnoses and surgical procedures made since 1977. We extracted data on participants' diagnoses entered into the registry during their first year of life. The diagnoses were coded according to the 8^th^ edition of the International Classification of Diseases (ICD-8) and as from 1994 according to the ICD-10 [23].

### Statistics

All data were entered into an EpiData database and exported to STATA for analyses. Student's t-test was used to test differences between continuous variables and for categorized variables; Chi-square test for independence within contingency tables was used. A two sided p-value below 0.05 was considered statistically significant. Analyses of the association between the exposure and the binary outcome variables were performed by use of simple logistic regression. The results are presented as odds ratios (OR) with 95% confidence intervals (CI). In addition, multiple logistic regression analysis was performed in order to analyze if gender, mother's age, maternal medical treatment, congenital abnormalities, preterm birth, and smoking should be considered confounders.

To evaluate the reproducibility of the method used, 10% of the children's dental charts were randomly chosen for re-examination by the first author. Kappa values were calculated to determine the presence of dental agenesis; the intra-examiner agreement was 100%.

## Results

A total of 214 exposed and 255 unexposed children were included in the study. The normality of the continuous variables was verified by histograms, and a significant difference between exposed and unexposed children's BW (p<0.05) and GA (p<0.01) was demonstrated (Table 2). In addition, the mothers in AED treatment group had a larger intake of analgesics and anti-psychotic drugs during pregnancy than the mothers not treated with AED. None of the potential confounders mentioned in the statistics section influenced, though, the risk for developing dental agenesis or other congenital abnormalities in case of prenatal exposure to AED.

**Table 2:** Characteristics of women-offspring dyads according to exposure or non-exposure.

	INCLUDED	
Number of children	Exposure (n = 214)	Non-exposure (n = 255)
Gender: boys/girls	114/100	120/135
Prescriptions^a^: Antimicrobials	95	94
Analgesics	21^d^	11
Antipsychotics	19^c^	6
Gestational age mean^b^ (SD)	39+1 (2+4)^c^	39+5 (1+5)
Birth weight, mean (SD)	3411 (656)^d^	3534 (553)
Smoking, n/total n (%)	76/203 (37)	85/248 (34)

^a^Prescriptions describe the number of women receiving a prescription during pregnancy.

^b^Gestational mean age is given as weeks + days.

^c^p<0.01.

^d^p<0.05.

The ORs for dental agenesis and other congenital abnormalities are shown in Table 3. Children exposed to AED *in utero* had an increased risk of developing dental agenesis, but as a group, the difference was not significant (OR = 1.7; [95% CI: 0.8–3.6]). Only a single child, which was from the exposed group, had agenesis of the similar primary teeth. The majority of the exposed children were either exposed to carbamazepine, oxcarbazepine, or valproate. The risk of developing dental agenesis was three-fold increased (OR = 3.1; [95% CI: 1.3–7.4]) in children exposed to valproate in mono- or in poly-therapy with other AEDs than carbamazepine or oxcarbazepine. The risk was further increased (OR = 11.2; [95% CI: 2.4–51.9]) in children exposed to valproate and carbamazepine or oxcarbazepine in combination (Table 3). Children exposed only to carbamazepine or oxcarbazepine, or exposed to other AEDs such as vigabatrin, rivotril, and lamotrigine, had no increased risk of dental agenesis whether they had been exposed to poly- or mono-therapy. Furthermore, children exposed to AED *in utero* had an increased risk of developing congenital abnormalities (OR = 2.0; [95% CI: 0.8–4.9]). The occurrence of congenital abnormalities increased significantly in case of prenatal exposure to carbamazepine or oxcarbazepine (OR = 3.1; [95% CI: 1.1–8.9]).

**Table 3:** The odds ratios and confidence intervals of congenital abnormalities according to whether the children were exposed or not during fetal Life (column 1) and according to whether the children were exposed to valproate,carbamazepine, oxcarbazepine, or other AEDs.

	Exposed	Valproate*	Carbamazepine or oxcarbazepine**	Valproate and carbamazepine or oxcarbazepine	Other AEDs in mono-therapy
Number of children	214	70	76	8	60
Dental agenesis, OR (95% CI)	1.7 (0.8–3.6)	3.1 (1.3–7.4)^a^	1.3 (0.5–3.8)	11.2 (2.4–51.9)^a^	-
Congenital malformations, OR (95% CI)	2.0 (0.8–4.9)	1.4 (0.4–5.4)	3.1 (1.1–8.9)^a^	-	1.6 (0.4–6.3)

*In mono- or poly-therapy other than carbamazepine and oxcarbazepine.

**In mono- or poly-therapy other than valproate.

^a^Significant p<0.05.

We found no significant differences in the number of congenital abnormalities between the exposed and unexposed children (Table 4). However, heart defects were registered in five of the exposed children and in none of the unexposed children. Furthermore, we found twice as many children with multiple congenital abnormalities in the exposed group. None of the children with proved dental agenesis were registered with any of the other congenital malformations.

**Table 4:** Number of children with the different congenital abnormalities according to whether they were exposed to antiepileptic drugs during fetal life or not.

	Exposed	Not exposed
Number of children, total	214	255
Eye and ear	2	2
Heart defects	5	0
Oro-facial clefts	1	0
Digestive system	1	0
Genital	2	1
Urinary	1	1
Bone and muscle	4	6
Chromosomal	1	0
Dental agenesis (%)	18 (8.4)	13 (5.1)
Congenital abnormalities (%)	14 (6.5)	8 (3.1)
Multiple congenital abnormalities (%)	4 (1.9)	2 (0.8)

## Discussion

Many studies have shown that children exposed to AED during fetal life have an increased risk of developing a variety of congenital malformations depending on the drug used and whether the child was exposed to mono- or poly-therapy [24], [25]. The present study shows that dental agenesis may arise as a potential congenital abnormality due to prenatal exposure to valproate. The risk of developing dental agenesis rose significantly if valproate was combined with either carbamazepine or oxcarbazepine.

The demonstrated lower BW and GA of subjects in the exposed group, the increased intake of prescription-based medication, and the distribution of the different congenital abnormalities are all in agreement with findings in large epidemiological studies [26], [27]. This agreement ensures the comparability and validity of our results. The prevalence of dental agenesis varies worldwide [7]. In Denmark, all dentists report dental agenesis in children to a central register with the National Board of Health [28]. In the year 2012, the prevalence was reported to be just below 4% based on a study population of 33,000 children [29]. This number is very similar to the findings in the larger international studies [7], [30]–[32]. In the present study, we found a prevalence of 5.1% in our control group, a figure that is within the variation demonstrated in the previous national and international studies.

Intrauterine exposure to valproate has previously been reported to induce an increased risk of spina bifida, heart defects, and cleft palate. Furthermore, a recently published study suggested an increased risk of jaw and mouth malformation due to the teratogenic effect of valproate [33]. Spina bifida, heart defects, and cleft palate are all congenital abnormalities in organs derived from the neural crest cells, which also are the origin of the cells involved in tooth development [34]. Two studies by Orup *et al*. showed contradictory results in relation to prenatal exposure to AED and the risk of dental agenesis [16], [17]. Both studies, however, comprised only a very small number of exposed children. In the first study, the exposed group comprised children prenatally exposed to different kinds of AEDs [16]. The study reported a three-fold increase in the prevalence of dental agenesis (3rd molar not included) compared with the general population. In the second study, Orup and coworkers only examined children exposed to phenytoin in either mono- or poly-therapy, and found no increase in the presence of dental agenesis [17]. This suggests that different pharmacological interactions are at play, in relation to tooth morphogenesis, as also indicated by the present study.

In the present study, none of the children with dental agenesis had any other congenital malformations diagnosed one year after birth. This might indicate differences in the biological mechanism controlling tooth morphogenesis compared to morphogenesis of other organs. Tooth morphogenesis is under highly genetic control during the whole pregnancy which increases the complexity compared to other developing organs. This complexity may imply that the tooth morphogenesis is more susceptible to external exposure.

The etiology of non-syndromic dental agenesis is unknown, and different theories have been suggested. A widely accepted theory suggests that dental agenesis is caused by a quantitative defect resulting in the lack of cells to support the development of the last tooth within each tooth-class [35], [36]. Another theory stated by Kjær suggests that dental agenesis stems from an incomplete neurological development [37]. It goes for both theories that the developmental defects occur in fetal life and that they may therefore be due to teratogenic exposure. Furthermore, it is hypothesized by Kulkarni and co-workers that dental agenesis is a result of different sensitivity in the expression of genes involved in the tooth morphogenesis and that certain stages of the tooth development are more vulnerable than others [38]. In an animal study they found that epilepsy like (EL) mice showed an increased risk of dental agenesis and that the process of development was initiated during but did not proceed beyond the cap stage. Perhaps this suggested genetic vulnerability can also be expressed in case of harmful exposure.

The mechanism underlying valproate-induced teratogenesis remains largely unknown. In a recently published review, it has been suggested that histone deacetylase (HDAC) is a direct target for valproate and that HDAC inhibitors alter Wnt signaling which induces an altered gene expression [39]. Wnt signaling is essential for tooth development, and modulation of the signaling leads to variation in tooth number [40]. Such a modulation may therefore be implicated in the development of dental agenesis. In an experimental study, it was suggested that prenatal exposure to valproate may down-regulate retinol binding protein-4, which induces hypervitaminosis A [41]. The toxicological and teratogenic effect of hypervitaminosis A is well-known, and it can induce cranio-facial malformations in case of prenatal exposure [42]. Accordingly, older experimental studies have shown that hypervitaminosis A can result in dental abnormalities including dental agenesis in mice [43], [44].

In accordance with findings in other studies, we found an increased, although not statistically significantly, risk of developing congenital abnormalities following prenatal exposure to AED. In general, congenital malformations occur infrequently compared with dental agenesis. This might be a reason for why we were unable to show any significant relation in the present study. However, the distribution of the malformations is in accordance with that reported by other studies; and more severe malformations like heart defects and spina bifida were all present only in the exposed group, while minor malformations like talipes equinovarus (club foot) were most common in the group of unexposed children. In addition, multiple congenital abnormalities were seen more often in the exposed than in the unexposed group. We found no association between dental agenesis and other congenital abnormalities.

Valproate is considered to be highly teratogenic compared to other AEDs and may result in a ‘fetal valproate syndrome’. The ‘fetal valproate syndrome’ has specific facial features, which are often diagnosed later than one year after birth; thus, we cannot exclude that some of the children may have an unrecognized ‘valproate syndrome’ [45]. Future studies would benefit from a wider time window for recording of congenital diagnoses, e.g. five years.

## Conclusions

Prenatal exposure to valproate as either mono or poly-therapy appears to be associated with dental agenesis. Dental agenesis should be considered a congenital abnormality that arises due to the teratogenic effect of valproate, and dental agenesis may be a sensitive marker of the teratogenicity of valproate.
